# Knowledge, attitude and practice (KAP) on food-drug interaction (FDI) among pharmacists working in government health facilities in Sabah, Malaysia

**DOI:** 10.1371/journal.pone.0304974

**Published:** 2024-07-11

**Authors:** Jackie Ho Chit Khong, Tuan Mazlelaa Tuan Mahmood, Sze Ling Tan, James Yau Hon Voo, See Wan Wong

**Affiliations:** 1 Faculty of Pharmacy, University Kebangsaan Malaysia, Kuala Lumpur, Malaysia; 2 Department of Pharmacy, Sabah Women and Children Hospital, Kota Kinabalu, Sabah, Malaysia; 3 Department of Pharmacy, Queen Elizabeth II Hospital, Kota Kinabalu, Sabah, Malaysia; 4 Department of Pharmacy, Duchess of Kent Hospital, Sandakan, Sabah, Malaysia; 5 Pharmaceutical Services Division, Sabah State Health Department, Kota Kinabalu, Sabah, Malaysia; University Putra Malaysia: Universiti Putra Malaysia, MALAYSIA

## Abstract

**Background:**

Food-drug interaction (FDI) is prevalent in our day-to-day life. Widely recognised as drug expert, pharmacists are responsible to provide patient education, including on FDI, to ensure optimum safety and efficacy of treatment. Most pharmacists have knowledge and experience regarding FDI to certain extent. However, the level of knowledge, attitude and practice (KAP) towards FDI among pharmacists were yet to elucidated for many countries, including for Malaysia.

**Objective:**

This study aims to assess pharmacists’ KAP on FDI, their inter-association, as well as association with sociodemographic characteristics in Sabah, Malaysia.

**Methods & materials:**

A cross-sectional study was conducted from 1 to 31 May 2023, using online, self-administered Google Form questionnaire, involving 24 government hospitals and 113 government health clinics in Sabah.

**Results:**

A total of 273 (or 35.5%) out of 768 pharmacists responded. Over two-third were female and serving government hospitals (79.6%). Mean score of KAP were 72.5 ± 14.3%, 93.2 ± 8.3% and 56.0 ± 16.7%, respectively, reflected good attitude but moderate knowledge and practice. Knowledge gaps identified include common medications such as paracetamol and metformin.Additionally, 28.2% respondents reported lack of FDI coverage during undergraduate, while merely 17.2% have received formal training on FDI after started working. Although 89.0% respondents agree that informing patients about possible FDIs is their responsibility, only 35.9% of the respondents usually or always practiced this. Such discrepancy maybe attributed to insufficient pharmacists’ knowledge on FDI, where pharmacists with good level of knowledge have almost 2 times higher odd for good level of practice, compared to those with poor/moderate knowledge (odds ratio, OR: 1.92; 95% CI 1.02–3.61; p = 0.040) in this study.

**Conclusion:**

There are significant training gaps to be filled in. Pharmacist possessing good knowledge is a prerequisite for better pharmacy practice. Adequate education strategies covering FDI should be emphasised for all pharmacy undergraduates and practising pharmacists.

## Introduction

Food-drug interaction is defined as an interaction resulting from a physical, chemical, physiologic, or pathophysiologic relationship between a drug and a nutrient, multiple nutrients, food in general, or nutritional status [1]. Specifically, FDI is defined as alteration of pharmacokinetics or pharmacodynamics of a drug or nutritional element (due to food), or a compromise in nutritional status as a result of the addition of a drug [2].

Food may affect pharmacokinetics of a particular drug by altering its disposition, which covers absorption, distribution, metabolism and elimination [3]. An example of FDI altering drug pharmacokinetics is the interaction between dairy product and ciprofloxacin, where taking both simultaneously will reduce absorption of ciprofloxacin due to formation of calcium-drug complex [4]. Neuvonen et al. 1991 showed that taking milk or yogurt reduced the peak plasma concentration, Cmax (by 36–47%) and bioavailability, AUC0-24h (by ~37%) of ciprofloxacin [5]. This was consistent with other studies [6–8]. Recognising the impacts, manufacturer recommends that ciprofloxacin tablet should be taken minimum two hours before or six hours after consuming multivalent cation-containing products, such as dairy products [9]. In this instance, as antimicrobial is involved, subtherapeutic level in serum or target organ may cause antimicrobial resistance [2], which is our priority to avoid in the current era of inadequate antibacterial clinical pipeline, as highlighted by World Health Organisation [10].

Another excellent example would be the interaction of halofantrine with fatty food [11, 12]. Halofantrine is an antimalarial. Milton et al. 1989 reported that after taken a fatty meal that contained about 60g fat, area under the curve or AUC (representing bioavailability) of halofantrine increased from 3.9 ± 2.6 mg/L/hr to 11.3 ± 3.5 mg/L/hr (by ~190%), while peak plasma concentration, Cmax of halofantrine increased from 184 ± 115 mcg/L to 1218 ± 464 mcg/L (by ~562%). Taking halofantrine with food promotes the release of bile acid, which enhances its absorption. This potentially leads to toxic halofantrine concentrations, increases risk of cardiotoxicity tremendously, which may cause arrhythmia and even cardiac arrest. Therefore, halofantrine should be taken on empty stomach to prevent adverse events.

On the other hand, drug pharmacodynamics is affected when both drug and food substance act on the same receptor or physiological system [3]. These interactions may be synergistic (where agents exert same pharmacodynamic effect, hence enhance the drug effect) or antagonistic (where agents exert opposite pharmacodynamic effect, hence attenuate the drug effect). For example, warfarin antagonises Vitamin K1 (phytomenadione) recycling by inhibiting Vitamin K epoxide reductase (VKOR), thus reduces the amount of active phytomenadione available for synthesis of blood clotting factors. On the other hand, taking green leafy vegetables that generally rich in Vitamin K1 opposes the effect of warfarin [13].

FDI is prevalent in our daily life, especially among patients with chronic medications [14]. This is because generally, the higher the number of medications consumed, and the longer the duration of the disease, the higher is the probability of FDI [14–17]. A few studies identified prevalence of FDI in their local population [14, 15, 18], where percentage reported was as high as 89.8% [15].

As the average lifespan of the population of Malaysia as well as of the world increases [19], so as the quality of healthcare, there is growing dependency on medications, especially for tackling non-communicable diseases and neoplastic diseases [20, 21]. Besides that, there are more medications available, which are definitely increasing not only in terms of number but also in terms of complexity [22, 23]. Therefore, it is inevitable that FDI will become increasingly more common.

In the United States of America, the annual expenditure for addressing drug-related morbidity and mortality amounts to approximately $37.6 billion, in which preventable medication errors, including FDI, constituted nearly half of this expense [24–26]. Inappropriate use of medications, including involvement of FDI, is associated with increased healthcare cost [27] because it may compromise treatment efficacy, safety or both, which in turn give rise to negative consequences such as worsening health condition, prescribing cascade, extended hospitalisation, and worst, death [15].

As the medication expert, while working in multidisciplinary team, pharmacist may play important roles by acting as gatekeeper to minimise potential prescribing or administration errors [28, 29], including FDI [30]. Pharmacists are also well positioned to provide education and support for other healthcare professionals (HCPs), so that these HCPs may assist in educating patients during their patient encounter [31]. Educated HCPs are also more equipped to help minimise FDI among patients that maybe dependant on them for drug administration. For example, a study in a teaching hospital in Tehran, Iran showed that with systematic education modules delivered by ward pharmacist to the ward nurses, total observations of wrong drug administration associated with FDI was reduced from 44.6% to 31.5%, with p < 0.001 [32].

Concomitantly, pharmacists are also responsible to provide patient education regarding their medications to optimise their pharmacotherapy safety and efficacy [33–35]. Previous studies have shown that patient’s knowledge and awareness on FDI is generally low, and pharmacists are well-positioned to fill in this gap [36–38]. The standard set by Joint Commission on Accreditation of Healthcare Organizations (JCAHO) in The United States of America outlines that patients must be educated with clear instruction on possible FDI to support their continuous healthcare requirements. Hence, it is imperative that pharmacists have good level of knowledge on FDI so that they can effectively identify patients with high risk of FDI and provide evidence-based, up-to-date counselling [39] regarding timing of medications with regards to mealtime, what food need to be avoided and other measures to minimise FDI [40].

In order to carry out this responsibility effectively, pharmacists themselves need to have sufficient knowledge [33]. Most pharmacists have knowledge and experience regarding FDI to certain extent. However, previous published studies in several countries have shown that pharmacists’ level of knowledge on FDI were generally poor to moderate, where scores reported ranged around 40–65% [18, 33, 38, 41–43].

On the other hand, there is a relative lack of studies that explored pharmacists’ attitude and practice on FDI. For attitude, two studies from Egypt and Lebanon reported good attitude level [42, 44], where the corresponding percentage reported were 78.8% and 95.3%, respectively. In terms of practice, Sultan et al. 2021 reported the practice level of healthcare professionals studied as moderate (53.0%), and linear regression showed that practice was significantly influenced by knowledge [42]. Good knowledge is a precondition for good practice [45]. Pharmacists’ poor practice on FDI counselling or education may be attributable to deficiency of knowledge regarding FDI [43].

Currently, the level of knowledge, attitude and practice towards FDI among pharmacists have been reported for a few Asian and African countries, however Malaysia is not in the list yet. Therefore, this study aims to fill in and benchmark for this gap. Moreover, the differences in healthcare system and pharmacy practice between Malaysia and these countries may render results different from the previous studies, hence it is imperative for Malaysia to have its own local study on this aspect.

## Materials and methods

This was a quantitative cross-sectional study. The study was conducted by convenience sampling, using self-administered Google Form questionnaire between 1 May 2023 until 31 May 2023.

According to information provided by Pharmaceutical Services Division, Sabah State Health Department, up until 7 December 2022, there were 768 pharmacists (including both provisionally registered and fully registered) working in the 24 government hospitals and 113 government health clinics in Sabah. Based on Raosoft online sample size calculator [46], assuming a 5% margin of error, a 95% confidence level, and a 50% response distribution, the recommended sample size for this study was 257. Allowing for 5% dropout, a final sample size of 270 was obtained. This study targeted all the pharmacists working in the sites as mentioned above.

Inclusion criteria were the study respondent must be a registered pharmacist (either provisionally or fully) with Malaysia Pharmacy Board, and was working in the government hospitals or government health clinics in Sabah during the study. After graduated from the recognised Bachelor’s Degree in pharmacy, the pharmacy graduates would enter a one-year training programme in recognised training facilities, which may be government hospital, government health clinic, private hospital, pharmaceutical industry company or university. During this year, they are provisionally registered with Malaysia Pharmacy Board, and hence recognised as Provisionally Registered Pharmacist (PRP), during which they will work under the guidance and supervision of qualified pharmacist. After passed both the training programme as well as Pharmacy Jurisprudence Examination, they may qualify as fully registered pharmacist. Incomplete questionnaire (less than 80% of the questions answered) was excluded from the final analysis.

To reach out to the individual pharmacists better, the link for the study was distributed to the pharmacists via e-mail to all Heads of Pharmacy of the respective health facilities in Sabah, which in turn, would help to cascade the link further to the subordinates in respective facilities. The link was also distributed by the investigators through WhatsApp to their respective contacts whom fulfilled the inclusion criteria. Respondent information sheets and informed consent were included at the beginning of the online survey form. After reading the information sheet, respondents’ agreement to click ‘Yes, I agree’ at the bottom of the information sheet and proceed further to the survey items indicated their consent to participate in the study. Respondents were informed about the strict confidentiality of their data and the use of their data anonymously for research purposes only. Respondents were given the option of withdrawing from the study at any time. Participation was entirely voluntary, with no monetary compensation.The questionnaire for this study consisted of 4 sections, namely sociodemographic data, knowledge, attitude and practice. Permission for use and adaptation of the knowledge section [41], as well as attitude and practice sections [42] had been obtained from respective authors prior to the study commencement.

Knowledge section consisted of 3 parts, where Part 2.1 (12 questions) tested on specific pairs of FDI available from literature, Part 2.2 (12 questions) tested on correct time of drug intake with regards to meal, whilst Part 2.3 (6 questions) tested on drug-alcohol interaction. Each correct answer scored 1 point, while each wrong answer or ‘I don’t know’ scored 0. The possible range of total score was 0–30. Classification of knowledge level was based on total knowledge score (TKS). TKS 0–14 (<50%) reflected poor knowledge, TKS 15–23 (50-<80%) reflected moderate knowledge, whereas TKS 24–30 (≥ 80%) reflected good knowledge [41].

Attitude section consisted of 1 part only, with 4 questions that evaluated the attitude of pharmacist regarding FDI and FDI-related practices. Each question was scored using 3-point Likert scale, ranging between agree, uncertain and disagree. The possible scoring for positively-stated statements ranged from disagree (1) to uncertain (2) to agree (3), while the possible scoring for negatively-stated statements ranged from disagree (3) to uncertain (2) to agree (1). The possible range of total score was 4–12. Classification of attitude level was based on total attitude score (TAS). TAS 4–5 (<50%) reflected poor attitude, TAS 6–8 (50-<75%) reflected moderate attitude, while TAS 9–12 (≥ 75%) reflected good attitude [42].

Practice section consisted of 3 parts, where Part 4.1 consisted of 4 questions with 5-point-frequency response scale ranged from never (0) to always (4) that explored how frequent pharmacist carry out FDI-related practices mentioned. Both Part 4.2 and 4.3 each consisted of one open-ended question, and were the only two questions optional to be answered in the Google Form. Only Part 4.1 was scored. Total practice score (TPS) was represented by total score for Part 4.1. The possible range of TPS was 0–16. Classification of practice level was based on TPS. TPS 0–7 (<50%) reflected poor practice, TPS 8–11 (50-<75%) reflected moderate practice, while TPS 12–16 (≥ 75%) reflected good practice [42]. The open-ended questions were not scored. Instead, they were subjected to thematic analysis. Codes were assigned according to different categories or themes. Two independent investigators validated the codes and themes identified.

### Validity and reliability analysis of the questionnaire

For content validation of the questionnaire, a total of 12 reviewers were recruited, namely 4 from academia, 3 from government hospitals, 3 from government health clinics and 2 from community pharmacies. Community pharmacists were also invited due to their expertise in food-supplement interaction, in drug-supplement interaction and to a certain extent, in food-drug interaction. Content validation was performed by using Item-Content Validity Index, I-CVI and Scale-level Content Validity Index, S-CVI, as described by Yusoff 2019 [47]. Face validity was established simultaneously as the reviewers were asked to rate on clarity, on top of relevance and importance. When the number of reviewers is nine or above, the corresponding minimum CVI value is 0.78 [48]. All I-CVI, S-CVI/Ave and S-CVI-UA values obtained in this study ranged 0.80–1.00, hence achieved the minimum CVI value.

The questionnaire was modified based on feedback from reviewers as well as discussion or consensus among research investigators. Generally, questions were reworded for better comprehension of the respondents. For knowledge assessment, all the questions from original questionnaire [41] were retained, except on the subsection of best timing for drug intake in regards to food. A few drugs that have contradicting or inconsistent recommendations (on the timing of intake in regards to food) were replaced with those that have consistent recommendations. The recommended timing for drug intake in regards to food is also potentially affected by dosage forms (tablet versus capsule) and formulations (controlled-release versus immediate-release). Therefore, the corresponding dosage form and formulation for each drug selected were specified to minimise respondents’ ambiguity when answering this subsection. In addition, for attitude assessment, out of the 8 questions available in the original questionnaire [42], 4 were removed because they were more knowledge-based, instead of attitude-based. For practice assessment, there was no deletion or switching of questions.

Pilot study involved 20 pharmacists working in Sabah government hospital and health clinics. Cronbach-alpha for knowledge, attitude and practice sections were 0.832, 0.736 and 0.758, respectively, hence attained the minimum acceptable threshold of 0.7 [49]. The result of the pilot study was not included in the actual analysis.

### Statistical analysis

All data gathered in the study were pooled and analysed with Statistical Package for the Social Sciences (SPSS) Statistic version 28.0 (IBM Corp., Armonk, New York). For all analysis, 95% confidence interval with p-value equal to or less than 0.05 is considered to be statistically significant. Categorical data were presented descriptively. Analysis of relationship between pharmacists’ knowledge, attitude or practice with sociodemographic data was performed with univariate logistic regression. If p-value was <0.25, the corresponding sociodemographic characteristics were further analysed using multi-variate logistic regression. Meanwhile, association between knowledge, attitude and practice of respondents were analysed using chi-square or Fisher Exact Test and univariate, binary logistic regression. To enable generation of 2x2 table, for each aspect of knowledge, attitude and practice, the number of respondents with poor and moderate level were summed together, grouped as poor/moderate group, and compared against those with good level.

### Ethical approval

Site approval was acquired from Sabah State Health Director. Ethical approval was obtained from Malaysia Medical Research & Ethics Committee, MREC (NMRR ID-23-00352-TNF) and Research Ethics Committee, Universiti Kebangsaan Malaysia (UKM PPI/111/8/JEP-2023-173) prior to the study commencement.

## Results

Out of 768 pharmacists targeted, a total of 273 pharmacists responded. None were excluded due to incomplete questionnaire, hence yielded a response rate of 35.5%. Their data were analysed in this study **(Table 1).**

**Table 1:** Baseline sociodemographic characteristics (n = 273).

Characteristics	Frequency, n (%) *[unless otherwise stated]*
**Age, year, mean (SD)**	31.5 (4.2)
**Gender**	
Female	184 (67.4)
Male	89 (32.6)
**Country where first pharmacy degree is obtained**	
Malaysia	234 (85.7)
Australia	13 (4.8)
United Kingdom	12 (4.4)
Indonesia	9 (3.3)
New Zealand	3 (1.1)
Egypt	1 (0.4)
Taiwan ROC	1 (0.4)
**Highest level of pharmacy education obtained**	
Bachelor’s degree	261 (94.9)
Master’s degree	14 (5.1)
PhD	0 (0.0)
**Years of work experience in pharmacy field, year, mean (SD)**	7.07 (3.90)
**Current workplace, type of facility**	
Government hospital	217 (79.5)
Government health clinic	56 (20.5)
**Current workplace, geographical division**	
West coast (Kota Kinabalu, Putatan, Penampang, Papar, Tuaran, Kota Belud, Ranau)	100 (36.6)
Interior (Keningau, Tambunan, Tenom, Nabawan, Sipitang, Beaufort, Kuala Penyu)	76 (27.8)
Sandakan (Sandakan, Beluran, Kinabatangan, Tongod)	50 (18.3)
Northern (Kudat, Kota Marudu, Pitas)	24 (8.8)
Tawau (Tawau, Kunak, Semporna, Lahad Datu)	23 (8.4)
**Current workplace, unit**	
Outpatient / Ambulatory	100 (36.6)
Ward / Clinical	66 (24.2)
Logistics	34 (12.5)
Inpatient	29 (10.6)
Drug Information	15 (5.5)
Management	14 (5.1)
Cytotoxic Drug Reconstitution (CDR)	4 (1.5)
Therapeutic Drug Monitoring (TDM)	3 (1.1)
Medication Therapy Adherence Clinic (MTAC)	3 (1.1)
Galenical / Manufacturing / Admixture	3 (1.1)
Total Parenteral Nutrition (TPN)	1 (0.4)
Nuclear	1 (0.4)
**Inclusion of FDI as part of the curriculum during undergraduate studies**	
Yes	196 (71.8)
No	77 (28.2)
**Experience of attended training which informed about FDI (after started working)**	
Yes	47 (17.2)
No	226 (82.8)
**Experience of encountering any FDI in practice**	
Yes	232 (85.0)
No	41 (15.0)
**Source of reference for FDI**	
Database: UpToDate	198 (72.5)
Peers (pharmacists)	140 (51.3)
Database: Micromedex	137 (50.2)
Database: MIMS	134 (49.1)
Database: Lexicomp	132 (48.4)
Database: Medscape	129 (47.3)
Scientific articles / journals	91 (33.3)
Knowledge from undergraduate study	73 (26.7)
Handbooks	60 (22.0)
Healthcare professional (other than pharmacists)	58 (21.2)
Social media (Facebook / Instagram / Twitter / TikTok / XiaoHongShu)	19 (7.0)
Textbooks	14 (5.1)
WhatsApp / Telegram / WeChat / FB Messenger	12 (4.4)
Newspapers	5 (1.8)
Others	2 (0.7)
Never refer for FDI	4 (1.5)

Frequency, n is followed by percentage out of total.

Abbreviation: FB–Facebook, FDI–food-drug interaction, IQR–interquartile range, ROC–Republic of China, SD–standard deviation

Over two-third were female and serving government hospitals (79.6%). Over 80% respondents received no training on FDI after started working, despite similar percentage encountered FDI in their pharmacy practice so far. In addition, a total of 71.8% respondents acknowledged FDI was part of their undergraduate studies. Out of the 39 pharmacists that graduated from oversea universities, 28 of them (71.8%) acknowledged FDI was part of their undergraduate studies.

Mean score of knowledge, attitude and practice computed in percentage were 72.5 ± 14.3%, 93.2 ± 8.3% and 56.0 ± 16.7%, respectively, reflected good attitude but moderate knowledge and practice **(Table 2)**.

**Table 2:** The frequency distribution of the respondents based on level of knowledge, attitude and practice, together with corresponding mean score.

Level	Knowledge	Attitude	Practice
	Frequency, n (%)	Frequency, n (%)	Frequency, n (%)
Poor	16 (5.9)	0 (0.0)	82 (30.0)
Moderate	152 (55.7)	9 (3.3)	143 (52.4)
Good	105 (38.5)	264 (96.7)	48 (17.6)
Mean Score ± SD	22.0 ± 4.23)	11.2 ± 0.99	8.97 ± 2.67
Mean Score ± SD (in %)	72.5 ± 14.3	93.2 ± 8.3	56.0 ± 16.7

Abbreviation: SD–standard deviation

For FDI knowledge assessment, looking at analysis of specific pairs of FDI, most pairs were scored above 60%. The three pairs that scored the lowest were digoxin-wheat bran (21.6%), followed by levothyroxine-cauliflower (26.4%) and levodopa-protein (43.2%). Regarding best timing of drug intake with respect to food, the only drug that scored less than 60% was isotrenitoin capsule. Meanwhile, for drug-alcohol interaction, again most pairs were scored above 60%, except for metformin-alcohol interaction that only scored 44.0% **(Table 3)**.

**Table 3:** The result of assessment of knowledge on food-drug interaction.

KNOWLEDGE					
**Knowledge on specific pairs of food-drug interaction**					
	**Statement**	**Frequency, n (%)**			**Frequency, n (%) of Correct Response**
		**Yes**	**No**	**I don’t know**	
1	Amiodarone can be taken with grapefruit juice	27 (9.9)	184 (67.4)	62 (22.7)	184 (67.4)
2	There is interaction between atorvastatin and grapefruit juice	187 (68.5)	37 (13.6)	49 (17.9)	187 (68.5)
3	Excessive cauliflower consumption affects the efficacy of levothyroxine	72 (26.4)	27 (9.9)	174 (63.7)	72 (26.4)
4	Caffeine consumption affects the efficacy of diazepam	185 (67.8)	16 (5.9)	72 (26.4)	185 (67.8)
5	Patients on warfarin can vary their intake of green leafy vegetables as they desire	11 (4.0)	262 (96.0)	0 (0.0)	262 (96.0)
6	Patient taking theophylline should avoid excessive coffee or tea	205 (75.1)	13 (4.8)	55 (20.1)	205 (75.1)
7	Milk affects the efficacy of tetracycline	244 (89.4)	10 (3.7)	19 (7.0)	244 (89.4)
8	Patients taking monoamine oxidase inhibitors (MAOIs) should avoid eating aged cheeses	167 (61.2)	6 (2.2)	100 (36.6)	167 (61.2)
9	Wheat bran affects the efficacy of digoxin	59 (21.6)	25 (9.2)	189 (69.2)	59 (21.6)
10	Protein-rich foods affects the efficacy of levodopa	118 (43.2)	17 (6.2)	138 (50.6)	118 (43.2)
11	Grapefruit juice can be safely consumed with all antibiotics	11 (4.0)	223 (81.7)	39 (14.3)	223 (81.7)
12	Patients on spironolactone should avoid taking food rich in potassium	235 (86.1)	23 (8.4)	15 (5.5)	235 (86.1)
**Knowledge on best timing of drug intake with respect to food**					
	**Drug**	**Frequency, n (%)**			**Frequency, n (%) of Correct Response**
		**On empty stomach**	**After meal**	**I don’t know**	
1	Carbamazepine IR Tablet	31 11.4)	210 (76.9)	32 (11.7)	210 (76.9)
2	Penicillin V Potassium Tablet	206 (75.5)	50 (18.3)	17 (6.2)	206 (75.5)
3	Isotretinoin Capsule	28 (10.3)	154 (56.4)	91 (33.3)	154 (56.4)
4	Pantoprazole CR Tablet	259 (94.9)	9 (3.3)	5 (1.8)	259 (94.9)
5	Alendronate Tablet	262 (96.0)	10 (3.6)	1 (0.4)	262 (96.0)
6	Indomethacin Capsule	9 (3.3)	229 (83.9)	35 (12.8)	229 (83.9)
7	Levothyroxine Tablet	251 (91.9)	21 (7.7)	1 (0.4)	251 (91.9)
8	Griseofulvin Tablet	32 (11.7)	193 (70.7)	48 (17.6)	193 (70.7)
9	Metformin IR Tablet	8 (2.9)	263 (96.3)	2 (0.7)	263 (96.3)
10	Calcium Lactate Tablet	22 (8.1)	246 (90.1)	5 (1.8)	246 (90.1)
11	Artemether and lumefantrine (Riamet) Tablet	25 (9.2)	217 (79.5)	31 (11.4)	217 (79.5)
12	Prednisolone Tablet	4 (1.5)	267 (97.8)	2 (0.7)	267 (97.8)
**Knowledge on drug-alcohol interaction**					
	**Drug**	**Frequency, n (%)**			**Frequency, n (%) of Correct Response**
		**Yes**	**No**	**I don’t know**	
1	Chlorpheniramine	200 (73.3)	32 (11.7)	41 (15.0)	200 (73.3)
2	Paracetamol	177 (64.8)	71 (26.0)	25 (9.2)	177 (64.8)
3	Metformin	120 (44.0)	80 (29.3)	73 (26.7)	120 (44.0)
4	Isoniazid	190 (69.6)	22 (8.1)	61 (22.3)	190 (69.6)
5	Warfarin	242 (88.6)	16 (5.9)	15 (5.5)	242 (88.6)
6	Methotrexate	175 (64.1)	17 (6.2)	81 (29.7)	175 (64.1)

Based on attitude section, more than 97% respondents agreed that undergraduate pharmacy curriculum should include more exposure to FDIs, and it is important to update pharmacists’ knowledge about potential FDIs. Moreover, 89.0% respondents agree that informing patients about possible FDIs is their responsibility. However, only 52.0% of the respondents perceived that pharmacists shall report adverse drug reactions (that arose due to FDIs) encountered in practice to the regulatory agency, while 35.2% were uncertain **(Table 4)**.

**Table 4:** The result of assessment of attitude on food-drug interaction.

ATTITUDE				
	**Statement**	**Frequency, n (%)**		
		**Agree**	**Uncertain**	**Disagree**
1	Undergraduate pharmacy curriculum should include more exposure to FDIs	265 (97.1)	8 (2.9)	0 (0.0)
2	It is important to update my knowledge about potential FDIs	272 (99.6)	1 (0.4)	0 (0.0)
3	It is not necessary to report any adverse drug reaction (ADR) due to FDIs which I encounter at work to the regulatory agency	35 (12.8)	96 (35.2)	142 (52.0)
4	Informing the patients about the possible FDIs is not my responsibility	17 (6.2)	13 (4.8)	243 (89.0)

Abbreviation: FDI–food-drug interaction

For the practice section, a total of 70.7% respondents prefer to check for the FDIs on their own, using handbook or database, which as pointed out in the sociodemographic section of this study, the most popular databases in descending order were UpToDate, Micromedex, MIMS, Lexicomp and Medscape. Nevertheless, only 35.9% of the respondents usually or always counsel/inform their patients about the possible FDIs they may encounter, while 44.7% only did this sometimes. Similar percentage distribution was observed for the question on whether the respondents ask their patients regarding the medications and food supplements or herbal remedies that their patients use or intend to use together **(Table 5)**.

**Table 5:** The result of quantitative assessment of practice on food-drug interaction.

PRACTICE						
	**Statement**	**Frequency, n (%)**				
		**Never**	**Rarely**	**Sometimes**	**Usually**	**Always**
1	I ask my patients about their medications (prescription / over-the-counter) and food supplements or herbal remedies they use or intend to use together	5 (1.8)	56 (20.5)	115 (42.1)	60 (22.0)	37 (13.6)
2	I counsel/inform my patients about the possible FDIs they may encounter	3 (1.1)	50 (18.3)	122 (44.7)	76 (27.8)	22 (8.1)
3	I refer to the drug information centre (DIC) pharmacist for checking certain FDIs	50 (18.3)	92 (33.7)	78 (28.6)	37 (13.6)	16 (5.8)
4	I use a handbook or database (e.g. Uptodate, Medscape, Micromedex) to check for FDIs	3 (1.1)	21 (7.7)	56 (20.5)	102 (37.4)	91 (33.3)

Abbreviation: FDI–food-drug interaction

For regression analysis between sociodemographic characteristics versus knowledge, attitude or practice, univariate followed by multivariate logistic regression showed that none of the characteristics were significantly associated with knowledge or practice. Nonetheless, it was demonstrated that hospital pharmacists have 4.37 times higher odds (95% CI 1.35–20.13; p = 0.035) for better attitude, compared to those in health clinic **(Table 6)**.

**Table 6:** Association between sociodemographic characteristics with knowledge on food-drug interaction.

Sociodemographic characteristic	Knowledge						Attitude						Practice					
	Univariate logistic			Multivariate logistic			Univariate logistic			Multivariate logistic			Univariate logistic			Multivariate logistic		
	COR	95% CI	p-value	AOR	95% CI	p-value	COR	95% CI	p-value	AOR	95% CI	p-value	COR	95% CI	p-value	AOR	95% CI	p-value
**Gender**																		
Female	1.00						1.00						1.00					
Male	0.65	0.38–1.11	0.114	0.67	0.38–1.16	0.152	0.23	0.06–0.94	0.041*	0.28	0.07–1.16	0.079	0.82	0.42–1.63	0.576	-	-	-
**Age**	1.06	0.99–1.12	0.066	1.16	0.97–1.39	0.094	1.00	0.85–1.17	0.969	-	-	-	1.02	0.95–1.10	0.621	-	-	-
**Country where first pharmacy degree was obtained**																		
Malaysia	1.00						1.00						1.00					
Oversea	1.76	0.89–3.49	0.104	1.58	0.78–3.19	0.206	0.57	0.11–2.85	0.494	-	-	-	1.03	0.43–2.49	0.948	-	-	-
**Highest level of pharmacy education**																		
Bachelor’s degree	1.00						1.00						1.00					
Master’s degree	1.76	0.60–5.16	0.306	-	-	-	0.41	0.05–3.57	0.422	-	-	-	0.77	0.17–3.57	0.740	-	-	-
**Years of work experience in pharmacy field**	1.04	0.98–1.11	0.188	0.89	0.74–1.08	0.235	0.99	0.84–1.17	0.905	-	-	-	1.01	0.94–1.10	0.723	-	-	-
**Type of facility**																		
Health clinic	1.00						1.00						1.00					
Hospital	1.61	0.85–3.06	0.145	1.56	0.81–3.00	0.186	5.22	1.35–20.13	0.016*	4.37	1.11–17.24	0.035*	2.52	0.95–6.70	0.064	1.96	0.71–5.43	0.196
**Geographical location of facility**																		
West coast	1.00						1.00						1.00					
East coast (Sandakan & Tawau)	0.89	0.47–1.67	0.700	-	-	-	0.98	0.21–4.48	0.971	-	-	-	1.28	0.60–2.72	0.522	-	-	-
Others (Kudat & Interior)	1.09	0.61–1.93	0.771	-	-	-	2.04	0.37–11.40	0.416	-	-	-	0.74	0.35–1.59	0.441	-	-	-
**Working unit**																		
Non-ward	1.00						1.00						1.00					
Ward	1.24	0.71–2.19	0.450	-	-	-	2.61	0.32–21.29	0.369	-	-	-	1.96	1.01–3.85	0.048*	1.64	0.82–3.29	0.163
**Inclusion of FDI during under-graduate studies**																		
No	1.00						1.00						1.00					
Yes	1.32	0.75–2.30	0.332	-	-	-	0.72	0.15–3.55	0.686	-	-	-	1.07	0.53–2.15	0.849	-	-	-
**Attended training on FDI after working**																		
No	1.00						1.00						1.00					
Yes	0.86	0.44–1.66	0.645	-	-	-	1.69	0.21–13.83	0.626	-	-	-	1.34	0.61–2.92	0.466	-	-	-
**Encountered FDI in practice**																		
No	1.00						1.00						1.00					
Yes	0.90	0.46–1.79	0.771	-	-	-	1.65	0.33–8.23	0.542	-	-	-	2.16	0.73–6.39	0.162	1.82	0.60–5.47	0.287

Abbreviation: AOR–adjusted odd ratio; CI–confidence interval; COR–crude odd ratio; FDI–food-drug interaction

* p-value <0.05 denotes statistical significance.

On the other hand, Fisher Exact test and univariate logistic regression showed that attitude was not significantly associated with knowledge nor practice. However, pharmacists with good level of knowledge have almost 2 times higher odd for good level of practice, compared to those with poor/moderate knowledge (odds ratio, OR: 1.92; 95% CI 1.02–3.61; p = 0.040) (**Table 7)**.

**Table 7:** Association between knowledge-practice, knowledge-attitude, and attitude-practice.

**Characteristics**			**Statistics**	**p-value**	**Statistics of univariate logistic regression**
	**Knowledge** ^**CS**^				
	Poor/Moderate	Good			
**Practice**					
Poor/Moderate	148 (54.2%)	77 (28.2%)	χ^2^ = 4.23 df = 1	0.040*	OR = 1.92 95% CI = 1.02–3.61
Good	24 (8.8%)	24 (8.8%)			
	**Knowledge** ^**FE**^				
	Poor/Moderate	Good			
**Attitude**					
Poor/Moderate	6 (2.2%)	3 (1.1%)		1.000	OR = 1.18 95% CI = 0.29–4.83
Good	166 (60.8%)	98 (35.9%)			
	**Attitude** ^**FE**^				
	Poor/Moderate	Good			
**Practice**					
Poor/Moderate	9 (3.3%)	216 (79.1%)		0.368	OR = 4.26 # 95% CI = 0.24–74.4
Good	0 (0.0%)	48 (17.6%)			

Each sample size, n is followed by % of total

Abbreviations: χ^2^—chi-squared statistics; CI—confidence interval; df–degrees of freedom; OR—odd ratio

CS: Chi-square

FE: Fisher Exact Test

# Haldane-Anscombe correction applied

* p-value <0.05 denotes statistical significance.

## Discussion

In this study, assessment of knowledge on specific drug-food pairs revealed that the question that scored the highest was on the interaction between warfarin and green vegetables. Similarly, high percentage was reported for good knowledge on the existence of warfarin-alcohol interaction. As an anticoagulant, warfarin carries a significant bleeding risk, also has a narrow therapeutic index and is known to interact with many drugs [50]. Therefore, it is unsurprising that it is extensively covered in local counselling guides for Malaysia pharmacists. Examples of the guides are Anticoagulation Medication Therapy Adherence Clinic (AC-MTAC) Protocol and Garis Panduan Kaunseling Ubat-ubatan Edisi Ke-3 [51, 52].

Globally, metabolic syndrome, includes diabetes mellitus is on the rise and is a serious worldwide public health problem. Based on International Diabetes Federation (IDF), globally, 9.3% (463 million) adults have diabetes in 2019. Without good curbing strategies, this figure is projected to increase to 10.2% (578 million) by 2030 and 10.9% (700 million) by 2045. Locally, Malaysia is leading in terms of diabetes rate in Western Pacific, one of the highest in the world, with diabetes prevalence rose from 11.2% in year 2011 to 18.2% in 2019, projected to rise further to 31.3% in 2025, if left unabated [53].

Considering that metformin is still the first-line for government hospitals and health clinics in Malaysia, as well as internationally [54, 55], it is alarming that metformin scored the lowest among all medications tested. Similar pattern was also observed in previous studies [32, 40], stressing the crucial need for all pharmacists to be educated on the significance of metformin-alcohol interaction. According to Lexicomp Drug Interactions [56], the interaction is assigned risk rating X, means the combination should be avoided. This is because ethanol may ameliorate the adverse effect of metformin, enhancing the risk of lactic acidosis [57, 58], which is potentially lethal without prompt treatment [59].

In Sabah, education on drug-alcohol interaction is prudent, considering that alcohol consumption is prevalent among Sabahans, especially among the indigenous. Malaysia National Health and Morbidity Survey 2011 reported that the national prevalence of current drinking and risky drinking were 11.6% and 23.6%, respectively. Risky drinking was more common among indigenous people of Sabah and Sarawak, with odds ratio, OR of 2.74 [60]. This is further supported by a more recent study in Keningau, the capital of Interior (Pedalaman) District in Sabah, where 80.2% of the participants recruited admitted having consumed alcohol, with 41% were stratified as having a high risk for hazardous alcohol use [61]. As drug-alcohol interaction also affects drug pharmacokinetics and pharmacodynamics [62–64], it is crucial for pharmacists to possess good knowledge on this aspect.

Overall, out of the 3 aspects studied in the knowledge section, the aspect of best timing of drug intake with respect to food scored the highest, while the average score for the other two aspects (i.e. knowledge on specific food-drug pairs and drug-alcohol interactions) were about 66%. For knowledge, none of the questions scored 100%. These collectively presented a potential knowledge gap to be filled, where more emphasis should be given on the coverage of these two aspects when devising pharmacy education strategies.

Less than three quarter of the respondents acknowledged that FDI was part of the curriculum during their undergraduate studies, regardless of the countries from where they have obtained their pharmacy bachelor’s degree. This suggests that inclusion of FDI in the university curriculum was depending on the individual university, rather than the country, highlighting the need for formal inclusion or consolidation of FDI in pharmacy undergraduate curriculum across all universities, thus a potential gap to be filled by academia. This is further supported by the finding from this study, where all respondents agreed that undergraduate pharmacy curriculum should include more exposure to FDIs, reflected pharmacists’ awareness to solidify foundation in this imperative aspect to ensure better preparation for future practice [65, 66]. For example, over a quarter of the respondents in this study applied the related knowledge from their undergraduate studies in actual practice.

Moreover, majority respondents received no formal training on FDI after started working. This gap highlighted the need to strengthen FDI coverage in current training module for all practicing pharmacists. This was attested by the fact that almost all respondents agreed that it is important to update pharmacists’ knowledge about potential FDIs. Previous studies have consistently reported poor knowledge on FDI among practicing pharmacists [18, 33, 38, 41–43] and pharmacy students [65, 66], highlighting the need to strengthen education in this aspect.

Teaching should not be limited to didactic lecture but rather may include simulations and mock presentations (which focus on common, significant FDI in practice), and the task to identify minimum one FDI during ward attachment. Interprofessional learning with undergraduates from other healthcare disciplines such as medicine and nursing maybe introduced too. These strategies provide effective emphasis and impart better understanding on FDI and its real-life application in clinical practice [67–69].

These findings were also consistent with finding from previous study, where exposure to FDI through incorporation of the of food and nutrition component in pharmacy undergraduate and postgraduate courses was associated with better knowledge towards FDI [42].

Additionally, this study also showed significant association between type of facility (i.e. government hospitals versus health clinics) and pharmacists’ attitude on FDI, which was unseen in previous studies. It should be noted that there is a lack of studies that compared pharmacists’ attitude on FDI across different care settings. Nevertheless, the high odd for better attitude among hospital pharmacists, compared to their clinic counterparts, was potentially attributable to greater need to report adverse drug reaction to National Pharmaceutical Regulatory Agency (NPRA), as well as to inform pertaining drug interactions to the individual patients, due to more frequent encounters. Compared to clinic (primary care) pharmacists, generally, hospital (secondary or higher care) pharmacists more often encounter and need to counsel more complex multimorbid patients. These patients are generally prescribed with wider range of medications, in terms of number as well as complexity, which commonly involved more sophisticated drug interactions [14, 70–72]. Hence, it is anticipated that hospital pharmacists were more likely to inform patients on pertaining FDI and also report ADRs, compared to primary care pharmacists [73, 74].

However, it was concerning that only approximately half of the respondents perceived that pharmacists shall report adverse drug reactions (that arose due to FDIs) encountered during their practice to the regulatory agency, which is Pharmacovigilance Section of NPRA, in Malaysia. According to the ADR guidelines published by NPRA, ADRs related to suspected FDI is listed as ADRs that should be given priority for reporting by healthcare provider [75]. Previous studies among hospital pharmacists reported inadequate knowledge as one of the barriers identified towards ADR reporting [76, 77]. This highlighted the need for awareness enhancement programme covering all pharmacists as ADR reporting is a crucial component of pharmacovigilance in order to promote patient safety [78].

Besides that, although majority of the respondents agreed that informing patients about possible FDIs is their responsibility, less than half usually or always did this in actual practice. This highlighted a discrepancy between attitude and practice, showing that attitude does not necessarily translate into practice, as demonstrated by an Egyptian study [42]. This maybe attributed to insufficient knowledge on FDI among the pharmacists, consistent with the findings from our study as well as previous studies [42, 43]. A systematic review exploring barriers towards pharmacists’ counselling on dietary and herbal supplements (DHS) identified lack of knowledge on DHS as one of the barriers. Generally, respondents expressed their awareness on their inadequate knowledge on DHS and on potential drug interactions associated with it. Such inadequate knowledge contributed towards their hesitation and decreased their confidence to provide counselling pertaining to DHS. They attributed the lack of knowledge on DHS to inadequate coverage in university curriculum as well as in workplace training [79]. Therefore, similarly, adequate training or education is crucial to enhance pharmacists’ knowledge on FDI [33]. Improving pharmacists’ knowledge were shown to translate into better practice by enhancing their confidence, willingness and skills to provide patients’ education, including counselling, which in turn will help to enhance patient’s knowledge [43, 79, 80]. A cross-sectional epidemiological study in Brazil reported that prevalence of FDI increases with poorer patient’s knowledge [14]. Improving patient’s knowledge on FDI will help to minimise FDI and associated adverse effects [38].

At the same time, time constraint and inadequate workforce in pharmacy were another potential contributor to the discrepancy between attitude and practice. Pharmacy management are responsible to ensure sufficient manpower [81, 82]. Effective risk stratification strategies should be implemented so that patients on drugs which are more prone to FDIs, especially those of greater severity or significance, would be prioritised for counselling [15, 39]. For example, the dispensing software maybe linked to drug interaction detection software to identify and flag such patients for counselling with pharmacist [83].

This study successfully elucidated the level of knowledge, attitude and practice on FDI among pharmacists in Sabah, Malaysia. To the best of our knowledge, this is the first study that explored the KAP on FDI among pharmacists in Malaysia.

As this study is only carried out in Sabah, one out of the 13 states in Malaysia, the finding may not necessarily be reflective of the level of KAP towards FDI among pharmacists from other states in Malaysia, however this study may serve as a very useful benchmark to plan future related studies for more comprehensive coverage on this crucial issue in Malaysia. For example, a nationwide study maybe employed to establish the national data for Malaysia. This would help to enhance the generalisability of the study findings. The study may also be expanded into other patient-facing pharmacy settings such as private hospitals, private clinics and community pharmacies nationwide to explore the influence of different settings on pharmacists’ KAP towards FDI.

Last but not least, the use of qualitative online survey may limit the scope of responses available. Two open-ended questions were asked in this study and respondents were allowed for free typing when answering them. However, not all respondents answered them. For those that answered, the answers given tend to be very short and generic. Unfortunately, no further probing or exploration of further depth, especially on certain intriguing answers was possible as the study has been carried out anonymously. More in-depth, related qualitative research maybe planned for better follow-up on the gap identified from current study.

## Conclusion

Pharmacists working in government health facilities in Sabah demonstrated good attitude but moderate knowledge and practice. Educational strategies should be emphasised on the inadequate aspects identified, such as specific food-drug pairs, certain drug-alcohol interactions, and the importance of ADR reporting, including those associated with FDI. Greater coverage on FDI in pharmacy undergraduate curriculum (across all universities) and in educational activities for working pharmacists, to ensure 100% coverage, which was relatively lacking in the current system.

Additionally, rather than good attitude, good knowledge was significantly associated with good practice. Therefore, adequate and effective training or education strategies is crucial to enhance pharmacists’ knowledge on FDI because this were shown to enhance their skills, confidence and willingness for engaging in better practice. Collaboration of pharmacists with dieticians, nutritionists, academicians, Malaysia Pharmaceutical Services Programme and related stakeholders in the provision of training and production of reputable references will ensure more successful education on this imperative topic.

## Supporting information

S1 File Questionnaire used in this study. (DOCX)
