# Supplementary material for: Knowledge, attitude and practice (KAP) on food-drug interaction (FDI) among pharmacists working in government health facilities in Sabah, Malaysia
Source: PLoS One. 2024 Jul 11;19(7):e0304974. doi: 10.1371/journal.pone.0304974 (PMC11239116; doi:10.1371/journal.pone.0304974)
Supplement: S1 File — (DOCX) [file pone.0304974.s001.docx]

**Supporting Information 1**

**Knowledge, Attitude and Practice (KAP) on Food-Drug Interaction (FDI) among Pharmacists Working in Government Health Facilities in Sabah, Malaysia**

**SECTION 1: SOCIO DEMOGRAPHICS**

Please indicate the following using a tick (✓):

1. Gender

⧠ Male

⧠ Female

2. Age (please state in the nearest integer): ______________years old

3. Country where first pharmacy degree is obtained:

⧠ Malaysia

⧠ United Kingdom

⧠ Australia

⧠ Indonesia

⧠ Other: Please Specify: _______________

4. Highest level of pharmacy education obtained (Kindly be noted that the 4-year Master of Pharmacy, MPharm qualification obtained from United Kingdom is regarded as Bachelor's degree in Malaysia):

⧠ Bachelor’s degree

⧠ Master’s degree

⧠ PhD

5. Years of work experience in pharmacy field (please state in the nearest integer): ____________years

6. Where are you currently practicing pharmacy?

⧠ Government Hospital

⧠ Government Health clinic

7. Your facility belongs to which division?

⧠ West coast (Kota Kinabalu, Putatan, Penampang, Papar, Tuaran, Kota Belud, Ranau)

⧠ Kudat (Kudat, Kota Marudu, Pitas)

⧠ Interior (Keningau, Tambunan, Tenom, Nabawan, Sipitang, Beaufort, Kuala Penyu)

⧠ Sandakan (Sandakan, Beluran, Kinabatangan, Tongod)

⧠ Tawau (Tawau, Kunak, Semporna, Lahad Datu)

8. Specifically, in which unit are you working currently?

⧠ Outpatient

⧠ Ward / Clinical

⧠ Inpatient

⧠ Drug Information

⧠ Logistics

⧠ Management

⧠ Cytotoxic Drug Reconstitution (CDR)

⧠ Therapeutic Drug Monitoring (TDM)

⧠ Other: Please Specify: _______________

9. My undergraduate course studies included FDI as part of the curriculum.

⧠ Yes

⧠ No

10. After started working, have you attended any training where you were informed about FDI?

⧠ Yes

⧠ No

11. Have you come across any FDI in your practice?

⧠ Yes

⧠ No

12. What is your source(s) of reference for FDI? (Choose all that applies)

Database: Lexicomp

Database: Medscape

Database: Micromedex

Database: MIMS

Database: UpToDate

Healthcare professional (other than pharmacists)

Knowledge from undergraduate study

Newspapers

Peers (pharmacists)

Scientific articles / journals

Social media (Facebook / Instagram / Twitter / TikTok / XiaoHongShu)

Textbooks

WhatsApp / Telegram / WeChat / FB Messenger

Never refer for FDI

Other, please specify: _______

**SECTION 2: KNOWLEDGE**

**Part (2.1). Knowledge on specific pairs of food-drug interaction**

For each statement, please choose the best answer based on your knowledge

| **I don’t know** | **No** | **Yes** | **Statement** |  |
| --- | --- | --- | --- | --- |
|  |  |  | Amiodarone can be taken with grapefruit juice | 1 |
|  |  |  | There is interaction between atorvastatin and grapefruit juice | 2 |
|  |  |  | Excessive cauliflower consumption affects the efficacy of levothyroxine | 3 |
|  |  |  | Caffeine consumption affects the efficacy of diazepam | 4 |
|  |  |  | Patients on warfarin can vary their intake of green leafy vegetables as they desire | 5 |
|  |  |  | Patient taking theophylline should avoid excessive coffee or tea | 6 |
|  |  |  | Milk affects the efficacy of tetracycline | 7 |
|  |  |  | Patients taking monoamine oxidase inhibitors (MAOIs) should avoid eating aged cheeses | 8 |
|  |  |  | Wheat bran affects the efficacy of digoxin | 9 |
|  |  |  | Protein-rich foods affects the efficacy of levodopa | 10 |
|  |  |  | Grapefruit juice can be safely consumed with all antibiotics | 11 |
|  |  |  | Patients on spironolactone should avoid taking food rich in potassium | 12 |

**Part (2.2). Knowledge about timing of drug intake with respect to food**

Please choose the best time to take each medication with respect to food

*IR = Immediate-release ; CR = Controlled-release

| **I don’t know** | **After meal** | **Empty stomach** |  |  |
| --- | --- | --- | --- | --- |
|  |  |  | Carbamazepine IR Tablet | 13. |
|  |  |  | Penicillin V Potassium Tablet | 14. |
|  |  |  | Isotretinoin Capsule | 15. |
|  |  |  | Pantoprazole CR Tablet | 16. |
|  |  |  | Alendronate Tablet | 17. |
|  |  |  | Indomethacin Capsule | 18. |
|  |  |  | Levothyroxine Tablet | 19. |
|  |  |  | Griseofulvin Tablet | 20. |
|  |  |  | Metformin IR Tablet | 21. |
|  |  |  | Calcium Lactate Tablet | 22. |
|  |  |  | Artemether and lumefantrine (Riamet) Tablet | 23. |
|  |  |  | Prednisolone Tablet | 24. |

**Part (2.3). Knowledge about drugs-alcohol interactions**

For each medication, please choose whether there is drug-alcohol interaction or not

-- “Yes” means drug-alcohol interaction **exists**,

-- “No” means **there is no** drug-alcohol interaction

-- “I don’t know” means you do not know the answer

| **I don’t know** | **No** | **Yes** | **Medication** |  |
| --- | --- | --- | --- | --- |
|  |  |  | Chlorpheniramine | 25. |
|  |  |  | Paracetamol | 26. |
|  |  |  | Metformin | 27. |
|  |  |  | Isoniazid | 28. |
|  |  |  | Warfarin | 29. |
|  |  |  | Methotrexate | 30. |

**SECTION 3: ATTITUDE**

- 1. Do you agree with the following statement?

|  | **Do you agree with the following statements?** | **Agree** | **Uncertain** | **Disagree** |
| --- | --- | --- | --- | --- |
| 1 | Undergraduate pharmacy curriculum should include more exposure to FDIs |  |  |  |
| 2 | It is important to update my knowledge about potential FDIs |  |  |  |
| 3 | It is not necessary to report any adverse drug reactions (ADRs) due to FDIs which I encounter at work to the regulatory agency |  |  |  |
| 4 | Informing the patients about the possible FDIs is not my responsibility |  |  |  |

**SECTION 4: PRACTICE**

4.1 Kindly answer the following questions:

|  | **Question** | **0**  **Never** | **1**  **Rarely** | **2**  **Sometimes** | **3**  **Usually** | **4**  **Always** |
| --- | --- | --- | --- | --- | --- | --- |
| 1 | I ask my patients about their medications (prescription / over-the-counter) and food supplements or herbal remedies they use or intend to use together |  |  |  |  |  |
| 2 | I counsel/inform my patients about the possible FDIs they may encounter: |  |  |  |  |  |
| 3 | I refer to the drug information centre (DIC) pharmacist for checking certain FDIs |  |  |  |  |  |
| 4 | I use a handbook or database (e.g. Uptodate, Medscape, Micromedex) to check for FDIs |  |  |  |  |  |

- 1. What actions would you take when you encounter a case of FDI?
  2. Give suggestions on how to improve the awareness on FDI among pharmacists.

**THANK YOU**
